# Accuracy of Physical Function-based Fall Risk Assessments Among Community-Dwelling Older Adults: An ROC Analysis

**DOI:** 10.53520/rhm2025.104142

**Published:** 2025-04-29

**Authors:** Estefania Zamarripa, Kworweinski Lafontant, David H. Fukuda, Jeffrey R. Stout, Ladda Thiamwong

**Affiliations:** 1 Institute of Exercise Physiology and Rehabilitation Science, University of Central Florida, Orlando, FL, USA;; 2 College of Nursing, University of Central Florida, Orlando, FL, USA;; 3 Disability, Aging, and Technology Cluster, University of Central Florida, Orlando, FL, USA;

**Keywords:** Screening, Fall Prevention, Balance

## Abstract

**Introduction:**

Physical function assessments, such as the Timed-Up-and-Go (TUG) and Short Physical Performance Battery (SPPB), are commonly used to evaluate fall risk among older adults, yet they may misclassify individuals. Center-of-pressure postural sway path length (PS) is a criterion for assessing fall risk, but it is unclear how TUG and SPPB compare. This study examined the sensitivity, specificity, and accuracy of these three assessments in classifying older adults as high or low fall risk.

**Methods:**

We conducted a cross-sectional study of 234 community-dwelling older adults (women=203, age=75.0±7.0 years, height=159±7.9cm, Body Mass Index=30.0±6.3kg/m^2^). Cut off values for SPPB (<7 out of 12), TUG (≥20 seconds), and PS (>30 cm) were used to categorize participants as high or low fall risk. McNemar tests were used to compare fall risk assessment between assessments.

**Results:**

Participants had a mean TUG time of 10.2±6.3 seconds, SPPB score of 8.7±2.3, and PS of 34.7±21.3cm. Both TUG (high risk=10, low risk=224; X^2^=95.9, p<0.001) and SPPB (high risk=40, low risk=194; X^2^=55.7, p<0.001) significantly differed in fall risk assessment compared to PS (high risk=115, low risk=119). Compared to postural sway, TUG had a sensitivity of 4.3%, specificity of 95.8%, and accuracy of 50.9%. Similarly, SPPB had a sensitivity of 23.5%, specificity of 89.1%, and accuracy of 56.8%.

**Conclusions:**

TUG and SPPB both demonstrated low sensitivity and accuracy, suggesting that they may be better as measures of physical function rather than fall risk. Clinicians may benefit from using PS or other criterion measures to screen for fall risk.

## Introduction

Older adults (≥65 years old) are increasingly susceptible to preventable falls, which are the leading cause of injury and injury-related deaths among this age group.^1^ Each fall has the potential of worsening physical function and increasing the risk of frailty beyond natural aging.^2^ To reduce the risk of falls among older adults and intervene in a timely manner, it is crucial to assess physical function and identify those at high risk of falls. Previous research has demonstrated a strong relationship between physical function and fall risk among older adults. While fall risk is multi-factorial, encompassing mental, social, psychological, and physical factors,^3^ physical function assessments typically gauge fall risk primarily via measures of strength, balance, muscle weakness, and walking capabilities.^4^ Common physical function assessments include the Timed-Up-and-Go (TUG), the Short Physical Performance Battery (SPPB), and center-of-pressure postural sway path length (PS). The TUG is a combined chair stand and gait speed test, walking to and from a 3-meter line at a normal pace.^5^ The SPPB consists of a 3-part performance test (static balance, gait speed, and chair stands) and provides a comprehensive assessment of physical function.^6^ PS provides clinicians with information regarding the distance traveled while swaying within a static stance using a force plate and is considered a criterion for assessing fall risk within a single assessment given that it is a direct measure of balance unlike the TUG and SPPB.^7,8^

All three of these assessments have been well-studied and commonly used in previous research to assess fall risk among older adults.^8^ These assessments have gained popularity in clinical practice as well, with the TUG and parts of the SPPB listed in the Stopping Elderly Accidents, Deaths & Injuries (STEADI) fall risk screening algorithm, which was created and promoted by the U.S. Centers for Disease Control and Prevention.^9^ Furthermore, a recent scoping review of clinical practice guidelines for fall risk assessment compiled 13 sets of guidelines from across the globe, with 12 of them specifically stating that gait, balance, and/or mobility should be included in fall risk assessments, lending to the relevance of the TUG, SPPB, and PS.^10^ Additionally, the TUG, SPPB, and PS all meet common criteria for clinical practicality by being quick to administer,^11–13^ especially given recent advances in technology that have increased the portability and accessibility of PS measurements.^7,14–16^

Although all three are valid assessments of physical function, PS is typically considered a criterion fall risk assessment if a multifactorial model is not being used.^17–19^ However, fall risk via PS may not be accessible to some clinicians, given the need for force plates and other technology. Lack of access to the criterion measure may negatively impact the quality of fall risk assessments that clinicians are able to provide to their older adult patients. While the SPPB and TUG offer a low-cost alternative to clinicians, it is unclear how those assessments compare to PS regarding fall risk classification. Therefore, the purpose of this study was to examine the sensitivity, specificity, and accuracy of the SPPB and TUG for classifying high and low fall risk compared to PS in a sample of low-income, community-dwelling older adults. We hypothesized that both the SPPB and TUG would present with at least 80% sensitivity and specificity.

## Scientific Methods

### Participants

We conducted a cross-sectional preliminary investigation, which was a part of a larger study focusing on combining technology-based body and mind interventions to prevent falls and reduce health disparities in low-income older adults.^20^ This study was approved by the University of Central Florida Institutional Review Board (STUDY00003206), pre-registered on ClinicalTrials.gov (NCT05778604), and carried out in accordance with the Declaration of Helsinki. We recruited 234 community-dwelling older adults (women, n = 203; men, n = 31) with an average age of 75.0 ± 7.0 years and an average Body Mass Index (BMI) of 30.0 ± 6.3 kg/m^2^. Participants were recruited from the greater Orlando, FL, area via fliers, local newsletters, exhibitions, and word-of-mouth. Participants were included if they were (i) aged ≥ 60 years, (ii) had low-income status, based on 2019 U.S. Census guidelines (Poverty Thresholds, 2020), (iii) were living independently, and (iv) were able to perform all three of the physical function assessments. Older adults living in care facilities (e.g., assisted living, skilled nursing, etc.) were excluded from participation. All participants provided written informed consent prior to the start of the study.

### Physical Function Assessments

PS was assessed using the BTrackS Balance System force plate and software (Version 7.5.5, Balance Tracking Systems, Inc, San Diego, CA, USA). The BTrackS plate was positioned adjacent to a sturdy piece of furniture or walker to reduce the risk of falling during the assessment. Participants stood upright on the plate with their feet in the pre-marked locations, approximately shoulder width apart, with their hands on their hips, head positioned forward, and eyes closed. This position was kept consistent as each participant completed four 20-second trials; the first trial was for familiarization, and the last three trials were scored with PS averaged between the three trials and reported in centimeters. After each trial, participants were allowed to open their eyes for 3–5 seconds before beginning the next trial. The BTrackS Balance System has demonstrated excellent test-retest reliability in assessing postural sway with the eyes closed among community-dwelling older adults.^21^

The SPPB was administered by trained research assistants using a phone application (SPPB Guide, Novartis Pharmaceuticals Corporation, Basel, Switzerland)^22^ to standardize the instructions, timers, and scoring for all participants. The SPPB began by assessing balance, in which participants were asked to maintain their balance in three different static stances for 10 seconds each: side-by-side, semi-tandem, and full-tandem. Lower body power was then assessed through a 5-repetition sit-to-stand assessment. Participants were asked to stand up and sit down in a sturdy chair with their arms crossed across their chest five times as quickly as possible. The last SPPB assessment was the gait speed test, in which participants began the assessment standing behind a marked line. Participants were instructed to walk at their normal walking speed to a line marked on the floor four meters in front of them. This gait speed test was completed twice, and the scores were averaged. Scores from the balance, sit-to-stand, and gait speed components of the SPPB were combined to determine their overall SPPB score according to previously published scoring criteria.^6^

For each TUG assessment, participants were instructed to stand up, walk at their normal pace to a line marked on the floor three meters in front of them, turn around, and walk at their normal pace back to the chair and sit down. A sturdy chair (i.e., stationary and non-collapsable) was used for each TUG assessment, with participants seated so that there was approximately 90° of knee flexion for both knees. A research assistant walked behind each participant to mitigate the risk of falling during the assessment. Participants were timed with a stopwatch and completed one trial, with the trial duration measured in seconds and used as their score.

### Fall Risk Classification

Evidence-based cut off values were used to categorize participants as a high or low fall risk for each physical function assessment. For the SPPB, participants with a score of 7 or lower out of 12 were classified as high risk.^6^ Participants with a value of 20 seconds or more for the TUG were classified as high risk; this cut-off was based on previous research that utilized an identical TUG protocol with older adults.^5^ PS had a cut off value of 30 cm or more, which was based on a median split of normative PS values for our predominant demographic (older women between 70 – 79 years of age) using the BTrackS device.^7,23^, specified *a priori* in the published protocol for the larger clinical trial,^20^ and used in previous research as a component of the Fall Risk Appraisal (FRA) matrix developed by our research team.^24,25^

### Statistical Analysis

All data were stored using a Research Electronic Data Capture database managed by the University of Central Florida.^26,27^ All statistical analyses were completed using jamovi version 2.5.6.^28^ A Kolmogorov-Smirnov test confirmed that all data were non-normally distributed (p < 0.001), and Levene’s test confirmed equal variances for each variable. McNemar tests were used to compare fall risk assessments between assessments, classifying participants as high or low risk. Sensitivity was calculated as [True Positives/(True Positives + True Negatives)], specificity as [True Negatives/(True Negatives + False Positives)], and accuracy as [(True Positives + True Negatives)/N].^29^ Descriptive data are presented as mean ± standard deviation. The threshold for statistical significance was set at p < 0.05.

## Results

**Figure 1.**
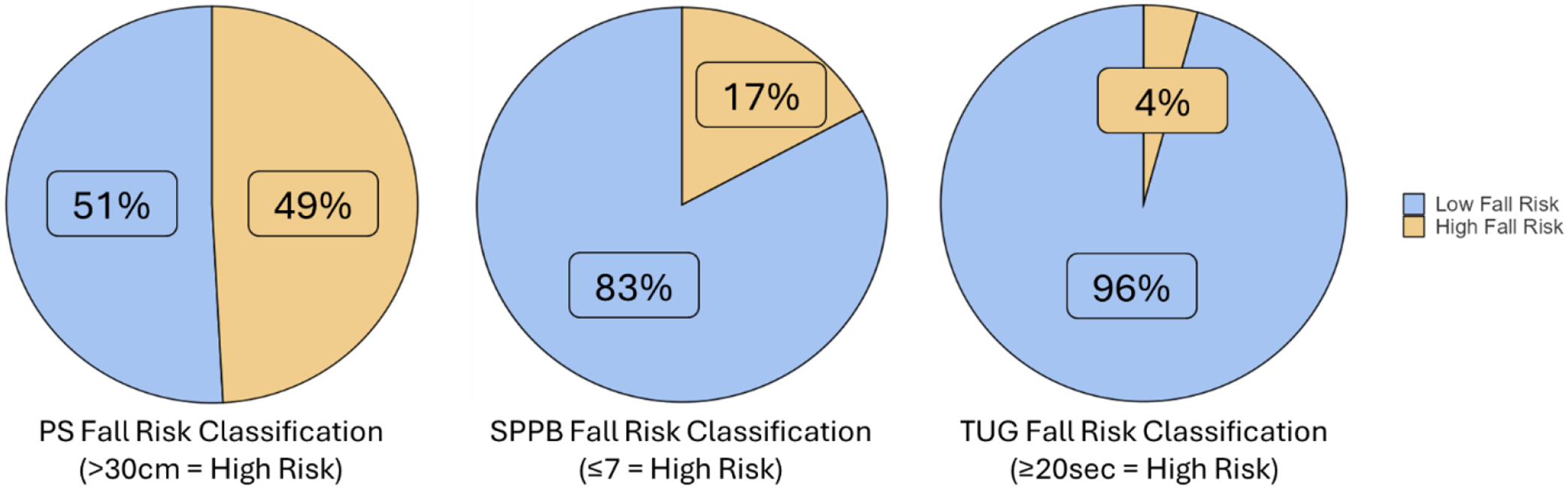
Fall risk assessment of postural sway (PS), the Short Physical Performance Battery (SPPB), and the Timed-Up-and-Go (TUG) with cut-off values of 30cm, 7 out of 12, and 20 seconds, respectively. N = 234.

After screening for inclusion criteria, 234 participants were included in the final analysis. Table 1 consists of demographic characteristics of the included participants. Figure 1 illustrates the fall risk classification for the TUG, SPPB, and PS. With the TUG, 10 participants were classified as high fall risk and 224 participants as low fall risk. With the SPPB, 40 participants were classified as high fall risk and 194 as low fall risk. As the gold standard, PS classified 115 participants as high fall risk and 119 as low fall risk.

Both TUG (X^2^ = 95.9, p < 0.001) and the SPPB (X^2^ = 55.7, p < 0.001) significantly differed in their fall risk assessment compared to PS. When compared to PS, TUG had a sensitivity of 4.3%, specificity of 95.8%, accuracy of 56.8%, positive likelihood ratio of 1.03, and negative likelihood ratio of 0.998. Similarly, SPPB had a sensitivity of 23.5%, specificity of 89.1%, accuracy of 56.8%, positive likelihood ratio of 2.15, and negative likelihood ratio of 0.859 compared to PS.

**Figure 2.**
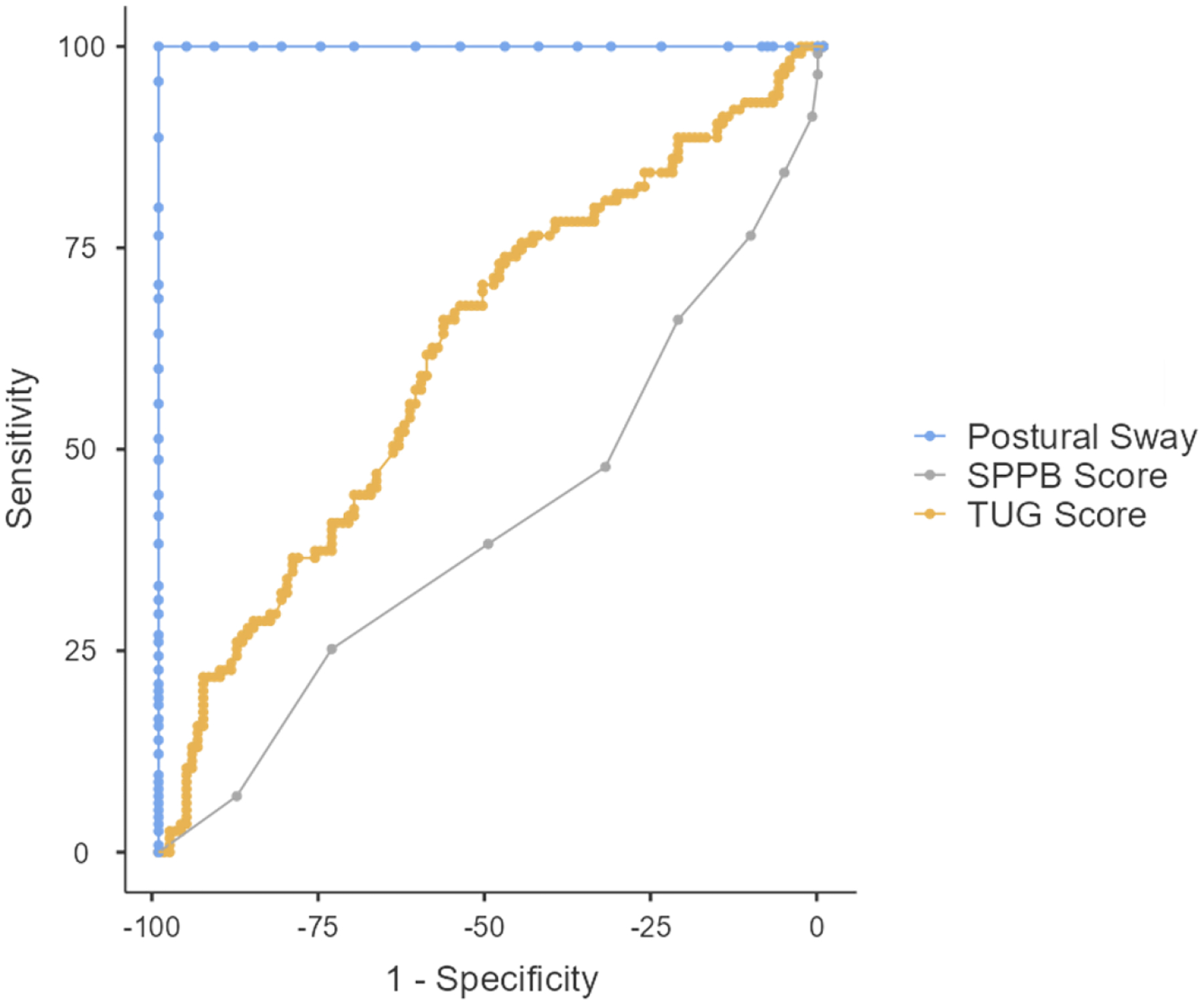
Receiver operator curve analysis of the Short Physical Performance Battery (SPPB) and the Timed-Up-and-Go (TUG) compared to PS as the criterion measure.

**Figure 3.**
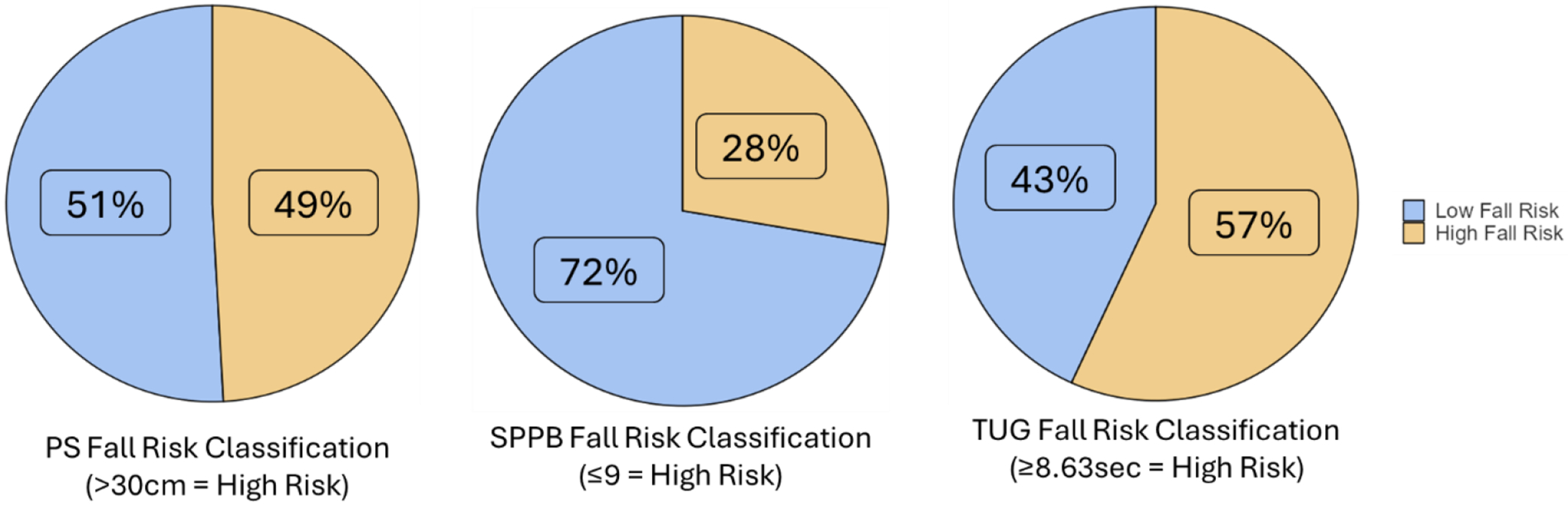
Fall risk assessment of postural sway (PS), the Short Physical Performance Battery (SPPB), and the Timed-Up-and-Go (TUG) with cut-off values of 30cm, 9 out of 12, and 8.63 seconds, respectively. N = 234.

While the focus of this manuscript was on categorical fall risk classifications for each assessment, we included a post-hoc receiver operator curve analysis for SPPB and TUG as continuous variables compared to PS (Figure 2). TUG had an area under the curve (AUC) of 0.621. The SPPB had an AUC of 0.412. We utilized the highest observed Youden’s Index (J) to determine new cut-off points for the TUG and SPPB (Figure 3). For the TUG, the highest observed J was at a time of 8.63 seconds (J = 0.224, sensitivity = 67.8%, specificity = 54.6%). This resulted in a classification of 133 participants as high fall risk and 101 participants as low fall risk, which were similar proportions when compared to fall risk assessment by PS (X^2^ = 3.52, p = 0.061). For the SPPB, the highest observed J was with a score of 9 (J = 0.194, sensitivity = 47.8%, specificity = 32.8%). This resulted in a classification of 169 participants as high fall risk and 65 participants as low fall risk, which was significantly different compared to classification by PS (X^2^ = 24.5, p < 0.001).

## Discussion

The purpose of this study was to examine the sensitivity, specificity, and accuracy in classifying high and low fall risk among low-income community-dwelling older adults between the SPPB and TUG compared to PS – the criterion measure. Contrary to our initial hypothesis of achieving at least 80% sensitivity and specificity for both tests, we found that fall risk classifications differed significantly from PS; the SPPB showed a sensitivity of 23.5%, and the TUG showed a sensitivity of 4.3%. On the contrary, specificity was relatively high (SPPB = 89.1%, TUG = 95.8%), indicating that both tools were better at identifying those at low rather than high risk. Although adjusting the TUG cut-off to 8.63 seconds yielded a fall risk distribution similar to PS, sensitivity and specificity remained unsatisfactory. Overall, these results suggest that the SPPB and TUG, at their established cut-off values, may help confirm low fall risk but fail to detect those older adults who are at high risk.

Pettersson et al. assessed 202 older adults using the TUG and SPPB against prospective fall incidence and found no association, with AUC values of around 0.5 – equivalent to random guessing.^30^ Our findings mirror this lack of predictive power, as we observed modest AUCs (SPPB = 0.412, TUG = 0.621) that similarly question the tests’ ability to identify high fall risk. One explanation for these results is that TUG and SPPB are highly task-specific: they evaluate performance under controlled conditions that may not reflect the complexity of daily life.^31^ This specific task could explain the low sensitivity we observed; subtle balance deficits that emerge under more challenging or unpredictable circumstances may go undetected. At the same time, their high specificity suggests that individuals truly at low risk generally perform well on these tests,^32^ reinforcing their utility as tools for confirming low risk rather than detecting high risk. Despite attempts to improve diagnostic accuracy by adjusting the cut-off values, neither the SPPB nor the TUG achieved our 80% sensitivity and specificity benchmark. Although the TUG revised cut-off (8.63 seconds) produced a distribution of risk similar to PS, the underlying diagnostic accuracy remained poor. The McNemar tests only compare the proportion of participants placed in each category, not where the unique participants are placed. These outcomes may have differed if PS was not set as the criterion, as prior studies used fall incidence rather than fall risk as the criterion measure.^30^ Notably, previous work by Lauretani et al. found significant associations between the SPPB and retrospectively reported falls,^33^ but the distinction between fall risk and fall incidence (both retrospective and prospective) may affect interpretability.

We selected PS as the criterion measure for classifying fall risk given that it is a direct assessment of the ability to maintain an upright posture, which is a key component of the definition of a fall.^34^ Unlike TUG and SPPB, which emphasize task completion time and functional abilities, PS quantifies the degree of postural sway, providing insight into underlying balance.^35^ An elevated PS indicates greater instability and potentially higher fall risk.^17^ By detecting subtle balance impairments that may not manifest during brief functional tests, PS may offer a more sensitive assessment of fall risk than the TUG and SPPB.

Although the TUG and SPPB are low-cost and widely accessible, their task-specific focus makes them fundamentally distinct from the static balance performance captured by PS. These differences likely explain why both tests failed to achieve strong diagnostic accuracy, regardless of the chosen cut-off. High specificity alone does not ensure clinical utility if sensitivity is too low to identify those who could benefit from interventions. From a practical standpoint, SPPB and TUG may be best viewed as initial screening tools to confirm low fall risk. Clinicians may need to pair the TUG and SPPB with another assessment to improve fall risk appraisals, as has been previously recommended for fall risk assessments.^36^ Similarly, PS may be a valuable addition to a multifactorial appraisal of fall risk, in line with previous recommendations.^37^

While this study was strengthened by the large and diverse sample of participants, several limitations must be considered when interpreting our results. Our use of a cross-sectional study design eliminated the ability to assess longitudinal accuracy in predicting fall incidence. Participants completed only one trial of the TUG, which may have affected their performance. Previous research with the TUG have varied in the number of trials used,^38^ and future research should work to determine if a learning effect is present with TUG performance when multiple trials are used. Additionally, both the TUG and SPPB were conducted at normal walking speed, which does not provide clinicians with any information on the maximal possible gait speed for each participant. While the use of a normal walking speed does mitigate the risk for falling during assessment, any potential technical breakdown in gait patterns would go undetected by clinicians if participants only walk at their normal speed, as technical breakdowns commonly occur at higher volitional efforts.^39^ Although the use of a normal gait speed is standard for the SPPB, gait speeds used for the TUG in previous research vary widely,^38^ which limits the ability to generalize the results of the present study to other work that utilized the TUG with a faster gait speed. The sensitivity, specificity, and accuracy of the TUG’s fall risk classifications may differ when a faster gait speed is utilized; although, more research is needed to test that theory. This study was also limited in its use of fall risk rather than fall incidence as a criterion factor, as fall incidence in the present study was too low to draw meaningful conclusions. However, fall incidence represents a different construct than fall risk and may not be interchangeable.

## Conclusions

While the TUG and SPPB are simple and quick to administer, their low diagnostic accuracy suggests that they are not interchangeable with PS as fall risk assessments. We observed low sensitivity and high specificity in classifying fall risk with the TUG and SPPB alone, which may not aid clinicians in correctly identifying older adults at high risk for falling. The accuracy of the TUG and SPPB may improve when using faster gait speeds; although, more research is needed to confirm that theory. Clinicians may benefit from combining the TUG or SPPB with another fall risk assessment to provide more information, thereby aiding in the correct fall risk classification of older adults.

## Figures and Tables

**Table 1.** Participants characteristics (N = 234)

Variable	Mean ± SD or n (%)
Age (years)	75.0 ± 7.0
BMI (kg/m^2^)	30.0 ± 6.3
Weight (kg)	76.5 ± 18.2
Height (cm)	159.0 ± 7.9
Sex	Male: 31 (13.2%)
	Female: 203 (86.8%)
Race/Ethnicity	African American: 83 (36.2%)
	Asian: 21 (9.2%)
	Hispanic: 84 (36.7%)
	Non-Hispanic White: 41 (17.9%)
TUG (seconds)	10.2 ± 6.33
SPPB	8.73 ± 2.30
PS (cm)	34.7 ± 21.3

**Note.** BMI = Body Mass Index; TUG = Timed-Up-and-Go; SPPB = Short Physical Performance Battery; PS = center-of-pressure postural sway path length.
